# The use of counting beads to improve the classification of fast breathing in low-resource settings: a multi-country review

**DOI:** 10.1093/heapol/czu047

**Published:** 2014-06-27

**Authors:** Aaltje Camielle Noordam, Yolanda Barberá Laínez, Salim Sadruddin, Pabla Maria van Heck, Alex Opio Chono, Geoffrey Larry Acaye, Victor Lara, Agnes Nanyonjo, Charles Ocan, Karin Källander

**Affiliations:** ^1^Health Section, United Nations Children Fund (UNICEF), Three United Nations Plaza, New York, NY 10017, USA, ^2^International Rescue Committee (IRC), 122 East 42nd Street, New York, NY 10168, USA, ^3^Save the Children, 501 Kings Highway East, Suite 400, Fairfield CT 06825, USA, ^4^UNICEF Supply Division, Oceanvej 10-12, 2100 København Ø, Denemarken, ^5^International Rescue Committee (IRC), Plot 7 Lower East Naguru Road, P.O. Box 24672, Kampala, Uganda, ^6^UNICEF Tamale Field Office, Norrip Building Complex, Bolgatanga Road, P.O. Box 1098, Tamale, Ghana, ^7^Population Service International (PSI), Whitefield Place, School Lane, Westlands, P.O. Box 14355-00800, Nairobi, Kenya, ^8^Malaria Consortium, Plot 25, Upper Naguru East road, P.O Box 8045, Kampala, Uganda, ^9^Department of Public Health Sciences, Karolinska Institutet, 17177 Stockholm, Sweden, ^10^Save the Children, 501 Kings Highway East, Suite 400, Fairfield CT 06825, USA, ^11^Malaria Consortium, plot 25, Upper Naguru East road, P.O Box 8045, Kampala, Uganda and ^12^Department of Public Health Sciences, Karolinska Institutet, 17177 Stockholm, Sweden

**Keywords:** Acute respiratory infection, case management, child health, community care, diagnosis

## Abstract

To decrease child mortality due to common but life-threatening illnesses, community health workers (CHWs) are trained to assess, classify and treat sick children. For pneumonia, CHWs are trained to count the respiratory rate of a child with cough and/or difficulty breathing, and determine whether the child has fast breathing or not based on how the child’s breath count relates to age-specific respiratory rate cut-off points. International organizations training CHWs to classify fast breathing realized that many of them faced challenges counting and determining how the respiratory rate relates to age-specific cut-off points. Counting beads were designed to overcome these challenges. This article presents findings from different studies on the utility of these beads, in conjunction with a timer, as a tool to improve classification of fast breathing. Studies conducted by the International Rescue Committee and Save the Children among illiterate CHWs assessed the effectiveness of counting beads to improve both counting and classifying respiratory rate against age-specific cut-off points. These studies found that the use of counting beads enabled and improved the assessment and classification of fast breathing. However, a Malaria Consortium study found that the use of counting beads decreased the accuracy of counting breaths among literate CHWs. Qualitative findings from these studies and two additional studies by UNICEF suggest that the design of the beads is crucial: beads should move comfortably, and a separate bead string, with colour coding, is required for the age groups with different cut-off thresholds—eliminating more complicated calculations. Further research, using standardized protocols and gold standard comparisons, is needed to understand the accuracy of beads in comparison to other tools used for classifying pneumonia, which CHWs benefit most from each different tool (i.e. disaggregating data by levels of literacy and numeracy) and what the impact is on improving appropriate treatment for pneumonia.

**KEY MESSAGES**

- The use of age-specific and colour-coded beads enables community health workers (CHWs) to track rather than mentally count the child’s respiratory rate and eliminates the need to remember the age-specific fast breathing cut-offs for pneumonia classification.
- Well-designed age-specific and colour-coded counting beads, when used in conjunction with an accurate timing device, have the potential to improve correct classification of fast breathing by CHWs with limited numeracy and literacy in low-resource settings.
- Further research is required to demonstrate the relationship between well-designed age-specific and colour-coded beads and health outcomes, particularly when data are disaggregated by key characteristics of CHWs (e.g. by levels of literacy and numeracy) and severity of illness.

## Introduction 

Because of the overall complexity of diagnosis, the still staggering mortality, lack of diagnostic aids and the growing problem of antibiotic resistance for pneumonia, there is an urgent need for more robust data on tools for pneumonia diagnosis. Pneumonia is the leading cause of childhood mortality, accounting for 17% of global deaths of children under five (UNICEF 2013). Timely recognition of pneumonia signs and symptoms, appropriate care seeking and access to antibiotic treatment can prevent many of these deaths. The Integrated Management for Childhood Illness (IMCI) protocol for ‘Caring for Newborns and Children in the Community’ guides community health workers (CHWs) to assess fast breathing as an indicator of non-severe pneumonia in children with cough and/or difficulty in breathing (WHO and UNICEF 2011).

There are two steps in detecting whether a child has fast breathing: (1) a CHW needs to visually count a child’s breath for 1 min and (2) the CHW has to determine how the child’s breath count relates to age-specific respiratory cut-off points (WHO and UNICEF 2011). International organizations training CHWs with various degrees of literacy and numeracy realized that many of them faced challenges in counting and relating the breath count to age-specific cut-off points. However, accurate assessment of fast breathing is crucial as selected children [children 2–11 months of age with a respiratory rate (RR) of 50 or more breaths/min and children 12–59 months of age with a RR of 40 or more] are classified as having pneumonia based on their breathing rate and require immediate treatment with antibiotics (Pio 2003; WHO and UNICEF 2005, 2011).

Watches and timers have been used as timing aids to facilitate 1-min RR counting. Box 1 shows an example of an acute respiratory infection (ARI) timer that is distributed by the United Nations Children Fund (UNICEF). Until now, there is limited evidence on counting devices and other affordable tools to help CHWs in resource-poor settings improve classification of fast breathing. One study evaluating the effectiveness of an abacus with a built-in sandglass concluded that CHWs were better able to correctly classify fast breathing with the breath counter (Bang and Bang 1992).

**Box 1 The ARI timer**

**Table:** 

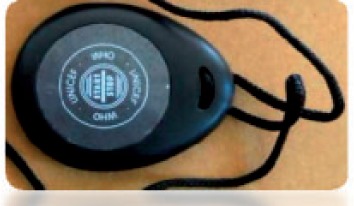	The ARI timer makes a ticking sound every second and has an alarm after 30 s as well as a final alarm to inform the user that 1 min has passed. The user must press the button to start the 1 min timing, during which a child’s breath is counted.

The potential of the use of counting devices, such as beads, is unknown due to lack of information regarding their effectiveness and utility. However, several organizations [including International Rescue Committee (IRC), Save the Children, Malaria Consortium, UNICEF and Population Services International (PSI)] have conducted small-scale studies among CHWs with differing levels of literacy and numeracy, using counting beads, which contribute to the knowledge base.

In this review, we compile the current evidence base of the effectiveness of counting beads to assess and classify breathing rates to guide pneumonia diagnosis. These findings are essential to further guide integrated Community Case Management (iCCM) programming aiming to decrease pneumonia deaths in young children.

## Methods

The findings presented are based on studies conducted by IRC, Save the Children, Malaria Consortium and UNICEF to improve iCCM programming in South Sudan, Uganda and Ghana. All CHWs in these studies were trained in iCCM using the World Health Organization (WHO) and UNICEF IMCI ‘Caring for Newborns and Children in the Community’ protocol. CHWs are named differently in various countries, e.g. in South Sudan they are called community-based distributors; however, in this article, we refer them as CHWs.

We compiled all research findings on the use of counting beads within these programmes. Although most findings presented are part of larger research initiatives, in this review we focused on the following questions:

- What is known regarding primarily illiterate CHWs’ ability to assess and classify fast breathing without the use of counting beads?
- Does the use of beads improve the ability of CHWs, particularly those with limited or no literacy and numeracy, to correctly classify fast breathing (hence, including tracking the breaths and classifying the breath count based on IMCI age-specific RR cut-off points)?
- Does the use of beads improve the ability of literate CHWs to correctly assess the breath count?
- What are CHWs’ perceptions of these tools, and does this differ by literacy level?

A brief description of the studies is provided below and a summary of the key methodological elements of the different studies can be found in Table 1. All studies used the ARI timer explained in Box 1, and the design of beads used by the various organizations can be found in Box 2. The results are separated by qualitative and quantitative research methods, as well as by organization. In addition, anecdotal evidence from two rapid assessments, from PSI in Democratic Republic of Congo and from IRC in Sierra Leone, is not included in the methods or findings, but is used to strengthen the discussion.

**Box 2 Overview of devices used by the various organizations**

**Table:** 

Picture	Description of the device	Used by
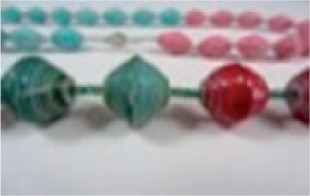	One set of two ‘age-specific and colour-coded’ strands of beads, with one bead counted per breath. The two strands can be distinguished because the beads of one age group have different colours and sizes than the beads of the other strand. Several tiny beads are used to create space between the beads, and the strand is tightly tied to hold the beads in place. At the end of age cut-offs for each strand, there are 10 differently colored (e.g. red/pink) beads which, if counted, indicate fast breathing. Strands have a clasp so they can be used open (straight) or closed (like a necklace). Beads are eclipse shaped and made from newspapers and glue.	Save the Children
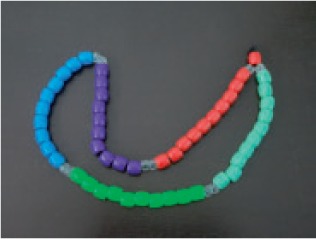	One strand of beads, non-specific for children ages 0–5 years and colour-coded per 10 beads to ease counting. The strand is necklace shaped and has a protruding start/end bead. One bead is counted per breath. Beads are made from plastic. CHWs are using these beads in conjunction with the ARI Timer. Some versions of the beads have an additional separating bead, between every bead.	Ministry of Health in Ghana and UNICEF
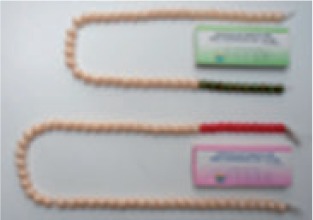	One set of two ‘age-specific and colour-coded’ strains of beads, with one bead counted per breath. The two strands can be distinguished because the 10 fast breathing beads match the age specific amoxicillin packaging used in Uganda. There are no separator beads, and they are tied so that there is space for moving the beads across the string. The beads are made from plastic and round shaped. Strands have a clasp so they can be used open (straight) or closed (like a necklace). The white beads are 16 mm in diameter, whereas the coloured beads are 12 mm in diameter.	IRC and Malaria Consortium
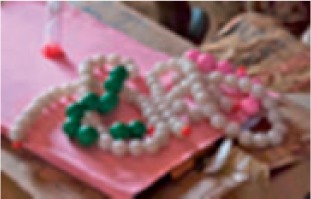	One set of two ‘age-specific and colour-coded’ strains of beads, with 1 bead/breath. The two strands can be distinguished because the fast breathing beads of one age group are different colors. There are no separator beads. The beads are made from plastic and round shaped. The strand is straight, with a small orange bead at the beginning and end.	PSI

**Table 1:** Summary of the key methodological elements of the different studies

	IRC		Save the Children US	Malaria Consortium	UNICEF	
Country	South Sudan	Uganda	South Sudan	Uganda	Uganda	Ghana
Geographic location	North Bahr el Ghazal: one sub-county in Aweil East	Karamoja: two sub-counties in Moroto district	Kapoeta North County, Eastern Equatoria	Bukomero, sub-county Kibonga	Kyankwanzi and Mpigi district	Tolon/Kumbungu and Central Gonja district
Year of research	2011	2011	2013	2012	2011	2012
Background characteristics						
Literacy level	100% illiterate	66% illiterate	100% illiterate	0% illiterate	0% illiterate	Mainly illiterate
Training iCCM	6 months prior	6 months prior	Part of research	Since 2010	>3 months prior	During 2007–2009
Use of ARI timer	6 months prior	6 months prior	Part of research	Since 2010	>3 months prior	During 2010–2012
Use of beads	Part of research	Part of research	Part of research	Part of research	Part of research	During 2010–2012
Type beads	Age-specific + colour coded	Age-specific + colour coded	Age-specific + colour coded	Age-specific + colour coded	Age-specific + colour coded	Non–age-specific and not colour coded
Quantitative research component						
Sample size	32 cases/32 CHWs	33^a^ cases/33 CHWs	69 cases/27 CHWs	282 cases/94 CHWs	Not applicable for this study (NA)	
Sample method	Random sampling	Random sampling	All CHWs attending the training	All CHWs included		
Assessing the impact	Timer vs timer and beads	Timer vs timer and beads	Timer and beads	Timer vs timer and beads vs mobile application		
Type and place of assessment	A mock visit at home of which the CHW was not aware, the children were not necessarily sick children	A mock visit at home of which the CHW was not aware, the children were not necessarily sick children	Selection of sick children at an out-patient department in a hospital	Video case scenarios (2 with fast breathing and one without)		
Correct using timer alone	Count within ± 3 breaths of gold standard + knew the cut-off point	Count within ± 3 breaths of gold standard + knew the cut-off point	NA	Count within ± 2 breaths of gold standard		
Correct using timer and beads	CHWs finger within ± 3 beads of gold standard	CHWs finger was within ± 3 beads of gold standard	CHWs finger in same classification area as gold standard	Count within ± 2 breaths of gold standard		
Qualitative research component						
Sample size	32 cases/32 CHWs	33^a^ cases/33 CHWs	NA	94 CHWs	87 CHWs	± 45 CHWs
Sample method	Random sampling	Random sampling		All CHWs included	Purpose sampling + snowball technique	Purpose sampling + snowball technique
Key research focus	Perceptions and potential improvements	Perceptions and potential improvements		Perceptions, user experience and opinions on communication of results	Perceptions and ideas for diagnostic aids	Perceptions, users experience and potential improvements
Method	Interviews	Interviews		Focus group discussions	Focus-groups, in-depth interviews and observations	

^a^46 were initially selected, but due to time constraints and the evident advantage of counting beads, the team stopped after assessing 33 CHWs.

### Quantitative research

#### IRC study in Uganda and South Sudan

As part of a larger assessment evaluating the quality of care provided by CHWs, IRC compared the CHWs’ ability to assess fast breathing using the ARI timer alone vs using the timer and beads. The geographical areas (payams) were selected on the basis of their geographical accessibility to the evaluation team and recent start-up of the programme.

First, CHWs were asked to describe the cut-off points for fast breathing for the two age groups (2–11 and 12–59 months) and to count the child’s RR using the ARI timer. A trained clinician who was part of the evaluation team counted simultaneously with the CHW. The RR from the clinician was written down and the CHW was asked to say his/her count. Afterwards, CHWs were given the right counting bead string for the age of the child being assessed and were shown how to move their fingers along the beads and to stop when the timer beeped. Then CHWs were asked to repeat the assessment with the same child using the timer and the beads. The clinician used the counting beads simultaneously with, but out of sight of, the CHW. The beads were counted back and recorded for both the CHW and the trained clinician. Microsoft Excel was used to analyse the data.

#### Save the Children study in South Sudan

The use of beads by CHWs with limited numeracy was part of an operational research lead by Save the Children. The research was conducted to assess the effectiveness of simulation-based training of CHWs using video technology.

During the training, CHWs were shown video clips of cases with danger signs (including case scenarios of malaria, pneumonia and diarrhoea) and the interactions between CHWs and caretakers. The video also showed the assessment, classification, treatment and advice on home care. CHWs were given one set (two strings) of beads to count and classify the breath count along with the ARI timer acting as a stop watch. CHWs were taken to a hospital outpatient department for post-training skills assessment, where they assessed sick children (40 out of the 69 cases had a history of cough). Each CHW managed (assessing, classifying and deciding to treat) two to three sick children aged 2–59 months. Each CHW also completed a knowledge questionnaire. As these particular CHWs were illiterate, one clinician trained in IMCI observed the CHWs’ management of sick children and recorded the assessment, classification and treatment findings on a study form. A senior evaluator who was also an IMCI trainer independently assessed the children at the same time and recorded his findings on a similar form. The RR was measured for all 69 children by the CHWs and the evaluator. To assess fast breathing the CHW had to choose the correct bead string for the age group. The observer marked the case as having fast breathing if the CHW reached the red beads (fast breathing for 2- to 11-month and 12- to 59-month age groups) within the minute. The evaluator used the ARI timer to count child breaths and noted the actual breath count in the form. The data were entered in CSPro and imported and analysed in Microsoft Excel.

#### Malaria Consortium study in Uganda

Malaria Consortium assessed whether there was a difference in accuracy in counting RR among CHWs using (1) the ARI timer alone vs (2) using the timer and beads and (3) using a mobile phone application, where the centre button on the phone was pressed for every breath observed, and which beeped after 1 min, after which the count was displayed. The sub-county was selected on the basis of its geographical location and mixture of CHWs with different age, sex and literacy levels [varying from being able to read and write well in any of the local languages (78%) to fairly well (22%)].

First, each CHW received a detailed explanation of how to count the RR, while simultaneously using the ARI timer, the same timer with beads as well as the mobile phone application. The CHWs then had the opportunity to practise three different methods of counting RR on videos of children with different breath counts. After familiarizing themselves with the three methods, each CHW was observed counting using the three options, respectively, on children with different breath counts. The video case scenarios were displayed on laptop screens, enabling one CHW to assess a child at a time. The final RR was recorded for the three tools as follows: (1) when using the timer alone, the CHW was asked for the final RR count, (2) when using both the timer and beads, the beads were counted back by a research assistant who provided the final RR count and (3) for the mobile phone application, the result was read from the screen. The breathing rate of the children in the video was known and was used as the gold standard. STATA 12 was used to analyse the data. The research only assessed the impact on counting the RR and did not assess the impact of these devices on classifying the breath count against the age-specific cut-off points.

### Qualitative research

Along with their quantitative research efforts, IRC and Malaria Consortium assessed CHWs’ perceptions about the beads, focusing on their opinions and proposed improvements. Data were collected through interviews. Malaria Consortium also assessed CHWs’ perceptions about how the use of counting beads helped them communicate the test results to caregivers.

Other qualitative research was initiated by UNICEF, as part of a broader effort to identify CHWs’ unmet needs regarding tools to support the assessment and classification of RRs. In Uganda the focus of the research was on CHWs’ experiences assessing RR using the ARI timer and their ideas regarding tools that might be useful to help improve their assessment and the classification of RRs. The CHWs were not familiar with the use of beads. Building on these findings, a subsequent study was conducted in Northern Ghana, where the CHWs had been trained to use both the ARI timer and beads. The main objective of this study was to help UNICEF improve the design of diagnostic aids based on the challenges CHWs indicated they face while assessing, classifying and identifying treatment needs for children under 5 with rapid breathing.

## Findings

Table 2 summarizes the key findings of various studies. In addition, an overview of the two different types of counting beads (a non–age-specific type and an age-specific type with colour-coded beads) can be found in Box 3.

**Box 3 Overview of the two types of counting beads intended for use in conjunction with an ARI timer**

**Non–age-specific counting beads**: These counting beads are designed to help the CHW keep track of the amount of breaths taken. The CHW counts moves bead for each breath. When 1 min has passed the CHW counts back the beads to determine the RR. Because of the colour coding of the number of beads (e.g. every set of 10), the CHW can count back the beads per colour: e.g. 1 colour = 10 breaths, 2 colours = 20 breaths, etc. On the basis of the RR, the CHW compares the result against the IMCI guideline, correctly remembering the age-specific cut-off rate.

**Age-specific beads with colour coding:** These counting beads support the CHW not only with counting but also with interpreting the RR against the IMCI guidelines. These beads remove the need to count by the CHWs to assess pneumonia (unless the actual RR is required for reporting purposes) because they consist of a set of two strands or rows of beads that are colour-coded to match the thresholds for the two different age groups. Depending on the age of the child, the CHW selects the matching bead strand and moves the beads for 1 min. Once the minute has passed, the CHW can identify whether the child has pneumonia, depending on the colour of the bead she/he is holding between his/her fingers.

**Table 2:** Summary table of key findings related to the four research questions

	IRC		Save the Children US	Malaria Consortium	UNICEF	
	South Sudan	Uganda	South Sudan	Uganda	Uganda	Ghana
1: What is known regarding primarily illiterate CHWs ability to ‘assess’ fast without the use of counting beads?	59% were not able to apply the age-specific cut-off points	46% were not able to apply the age-specific cut-off points	Before the use of beads, CHWs were not able to assess fast breathing as they could not count beyond 10 and do simple arithmetic	No specific data for this research question (NA)		
	72% made an incorrect count using the timer	33% made an incorrect count using the timer				
2: Does the use of beads improve the ability of CHWs, particularly those with limited or no literacy and numeracy, to correctly ‘classify’ fast breathing	The use of beads increased the correct classification from 13% to 63%	The use of beads increased the correct classification from 37% to 73%	The use of beads enabled 60% of the CHWs to correctly classify fast breathing			
3: Does the use of beads improve the ability of literate CHWs to correctly ‘assess’ the breath count?	NA			CHWs were 5.6 times more likely to count the RR correctly using the timer alone compared with when it is combined with beads	NA	
4: What are CHWs’ perceptions of these tools, and does this differ by literacy level?	Beads simplifies the classification of fast breathing		NA	Use of beads in made it easier to communicate the results to the caretaker	CHWs who only used the timer revealed that it would be useful to have a device to support them with classifying and communicating results to caregivers	CHWs used ‘non–age-specific beads’ and were still challenged in counting and remembering cut-off points
	Colour-coded beads match the locally available amoxicillin package, which helped CHWs identify appropriate strings					
				Combining the timer and beads is more tasking, with a slow bead movement process that reduces the accuracy		
	There was a request for more training and practice					
					Beads were perceived as easily confused with a toy, but useful	When shown ‘age-specific and colour-coded’ beads used by Save the Children and IRC, these were preferred

### Primarily illiterate CHWs’ ability to count breaths and their knowledge of age-specific cut-off rates

The IRC assessed what the key challenges were for primarily illiterate CHWs while classifying fast breathing. Findings indicated that 46% (21/46) of CHWs in Uganda were not able to apply the age-specific cut-off points and 33% (15/46) made an incorrect count using the ARI timer. In South Sudan, 59% (19/32) of CHWs were not able to apply the age-specific cut-off points and 72% (23/32) of CHWs made an incorrect count using the ARI timer.

### Primarily illiterate CHWs’ ability to classify fast breathing using counting beads

**Figure 1:**
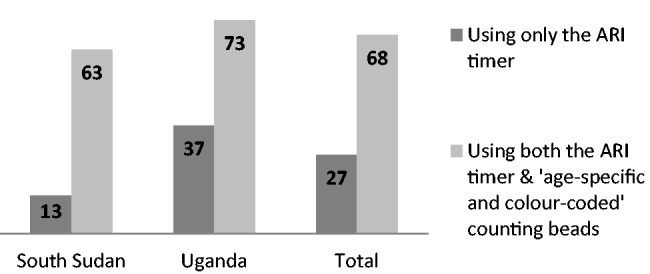
Percentage of CHWs able to classify fast breathing correctly, based on findings from IRC

In South Sudan 13% (4 out of 32) of the CHWs were able to classify fast breathing using only the ARI timer, whereas this number increased to 63% (20/32) (Odds Ratio (OR) OR = 11.7, *P* = 0.002) when they used the beads together with the timer. Findings were similar in Uganda, where the ability to classify fast breathing increased from 37% (17/46), using only the timer, to 73% (24/33) (OR = 4.4, *P* < 0.005), using both tools. Combining the data from both countries, the ability to classify fast breathing increased from 27% (21/78) to 68% (44/65) (OR = 5.7, *P* < 0.005), see Figure 1.

Save the Children’s research did not have a component of assessing a sick child without the use of beads, as they identified that CHWs had limited numeracy, and were not able to count beyond 10. A total of 69 sick children were assessed by the 27 CHWs. Using the ‘age-specified and colour-coded’ beads and ARI timer, the CHWs classified 25 cases as suffering from fast breathing. The senior evaluator who only used the ARI timer classified 23 as fast breathers. Of the 25 CHW-classified fast-breathing cases, 15 (60%) matched with the evaluator-classified fast-breathing cases.

CHWs were also administered a knowledge questionnaire that included questions on the use of beads appropriate for age. All 27 CHWs picked the beads appropriate for the two age groups. With respect to classifying a child as having fast breathing, 26 out of 27 picked the right colour (red) for the 2- to 11-month group and 27 out of 27 for the 12- to 59-month age group.

### Literate CHWs’ ability to correctly count breaths using counting beads

In Uganda, Malaria Consortium assessed whether the use of tools, including counting beads, improved the ability of literate CHWs to count RR. CHWs were 5.6 times more likely to count (not classify) RR correctly (i.e. ±2 breaths) using the timer alone compared with when it is combined with beads (OR = 5.6, *P* < 0.001). There was no significant difference between the ARI timer and mobile phone application (OR = 1.1, *P* = 0.08), implying that CHWs have a similar capacity to correctly count RR using either of the assessment methods (i.e. counting themselves vs pressing a button for every breath observed).

Overall, the median difference between the ‘true rate’ and the rate observed with the three methods was −1 (interquartile range(IQR) −5 to 2) for the UNICEF timer, −1 (IQR −7 to 2) for the mobile phone application and −5 (−12 to 2) for the ARI timer with beads. Using the sign test for non-parametric data on matched pairs, the differences in rates observed using the ARI timer compared with the true rate was not significantly different from 0 (*P* = 0.01), whereas for the mobile phone application and counting beads, the difference between the rates observed and the true rate was significantly different from 0 (*P* = 0.001 and *P* < 0.0001, respectively). Using the same test, the median difference observed between the ARI timer and mobile phone application was not significantly different (*P* = 0.179), whereas the difference was significantly different between the ARI timer and the counting beads (*P* < 0.001) as well as between the mobile phone timer and the counting beads and timer (*P* = 0.001).

When analysing the accuracy of the three different methods by the characteristic of the rate of the child in the video (i.e. normal or fast), it was demonstrated that all three methods performed much better on the slower breathing rates (i.e. 40 breaths/min) than the fast rates (i.e. 65 and 66 breaths/min). All three methods tended to overestimate the rate in the slow-breathing scenario, whereas they underestimated the rate in the fast-breathing scenarios.

### CHWs’ perceptions on the use and design of age-specific and colour-coded beads

The colour-coded beads designed by IRC in Uganda match the colour of the locally available amoxicillin packages for specific age groups, and according to (mainly illiterate) CHWs, this helped them to identify the correct string needed for the child, eliminating the need to recall the cut-off points for the different age groups. Regarding the use of counting beads, CHWs interviewed by IRC were most likely to say that: (1) the use of beads eliminated the need to count or worry about forgetting the number or making mistakes while counting; (2) it was easy to move hands along the beads; (3) it was easy to know when to give the treatment and (4) it was easy to explain to the mother that her child did not need medicine. The need for more training and practice regarding the use of beads in combination with the ARI timer was mentioned by CHWs in South Sudan and Uganda.

In the study amongst literate CHWs by Malaria Consortium in Uganda, CHWs expressed their support for alternative methods to count the RR other than the timer which they were familiar with. Using the beads, in addition to the timer, was perceived as being advantageous because each breath was being represented by movement of a bead and then counting of the beads could be done afterwards, thus giving more accurate results.

What I have liked about this method is that I don’t have to count the beads as I move them, counting usually comes last and this gives me more concentration on observing the child and moving the beads.

The method was acknowledged as one that could give a quick picture of the diagnosis by identifying the colour of the bead where the hand has stopped after the alarm has gone off (i.e. whether white or red/green bead), the next steps could follow later:

From what I have learnt today, the combination of both the timer and beads is very helpful because it helps me to immediately know whether the child has fast breathing by looking at the colour of beads where I have stopped and then, I count the beads to confirm what I have seen, after which I write in my book.

The use of the beads also made it easier to communicate the results to the caretaker, as the colours visually flagged if the child had a high breath rate (indicating pneumonia) or not.

However, the beads were also perceived by some as disadvantageous because combining the timer and beads was considered to be more tasking, with a slow bead movement process and therefore reduced accuracy. During observations, it was noted that CHWs often found it difficult to start moving the beads immediately after starting the timer.

For me, I find this method very challenging because I have to observe three things at the same time: I have to look at the child, start the timer and also move the beads at the same time, which is a bit tasking. That is why you have seen that I have been forgetting to put on the timer as I move the beads.

Related to the design, CHWs often mentioned that the space between individual beads can affect the outcome of the count. The space between the beads should be small, as the bigger the distance was between the beads, the more difficult it was to move them. The CHWs also suggested that the beads should be light and unattractive in order not to be mistaken for accessories and have a strong string, which would not break easily.

Data from the assessment in Uganda by UNICEF, where mainly illiterate CHWs only used the ARI timer, revealed that CHWs would find it useful to have a device that supports them with the classification of fast breathing and that would help them communicate the findings to the caregivers. When the CHWs in Uganda were shown counting beads they mentioned that beads might be confused with a toy; however, it would help them with the assessment and the classification of fast breathing.

I would have preferred the beads because each time I count, I hold a bead so I will be able to know how many beads I left behind in case I forget where I was when counting

Green is OK. Red is for danger.

### CHWs’ perceptions on the use and design of non–age-specific beads

The main concern among mainly illiterate CHWs regarding the use of non–age-specific beads used in the Northern region of Ghana was that these beads still required counting and remembering the cut-off rate. The beads also have ‘separator bead’, which the CHWs thought creates confusion because ‘we move the beads without looking at them’. Nevertheless, the use of prayer beads in the region is common; CHWs are used to counting with beads and are at ease with this. However, the CHWs reported that this similarity could reduce the acceptance by caretakers as the beads are perceived as a tool for prayer and not as a healthcare-related tool.

When shown the ‘age-specific and colour-coded’ beads used by IRC and Save the Children, the CHWs and their supervisors in Ghana showed a strong preference to this design as it eliminated the need to count and remember the cut-off rate.

This one has only two colours and the colours easily tell if the child has pneumonia or not. I think that this one will be more convenient to use than the current one we have.

## Discussion

This review of studies on the utility of counting beads as a tool to improve classification of fast breathing in children with cough and/or difficulty breathing to guide pneumonia diagnosis shows that the introduction of ‘age-specific and colour-coded’ counting beads, in addition to an accurate timer, can help CHWs with limited numeracy and literacy to more accurately assess fast breathing. Although CHWs also expressed concerns on the task intensity of the method, in general, it was acceptable and applicable across different settings.

There are several limitations associated with the studies included in this review. First, the studies used several research approaches, methods (including gold standards) and research questions. Moreover, programmatic settings as well as the levels of literacy and numeracy of the CHWs differed. Second, the case scenarios by the various organizations assessed children in different settings (at home, hospital or on a video screen), which resulted in different breathings patterns, e.g. the children assessed at home were unlikely to have fast breathing, although a few of them happened to have it. This review suggests that these factors influence the utility of beads.

Data from IRC, Save the Children and anecdotal evidence from PSI and IRC suggest that ‘age-specific and colour-coded’ beads enable and improve accuracy in the classification of fast breathing by CHWs with limited literacy and numeracy. However, if literacy and numeracy is not an issue, findings from Malaria Consortium show that the use of beads complicates the assessment, as it resulted in more inaccurate counts (i.e. CHWs were 5.6 times less likely to count correctly using the beads and timer). A reason for the breathing count inaccuracy by literate CHWs was that some perceived the use of beads as more task intensive. Although for CHWs with limited literacy and numeracy (e.g. those not able to count beyond 10), the use of beads enabled them to track the breathing rates as well as classifying the count against the age-specific cut-off points, which would not be possible without these beads.

It was also found that the rate of breathing influences the accuracy of the RR count. The research conducted by Malaria Consortium shows that, regardless of the tool CHWs used, they tended to overestimate the rate in the slow-breathing scenario, whereas they underestimated the rate in the fast-breathing scenarios.

### Recommendations

Regarding the use of counting beads, these data show that it is premature to conclude to which degree the beads, in addition to a timer, would improve the ability of trained CHWs to correctly classify fast breathing. A more conclusive assessment is needed amongst sick children, disaggregating data by intensity of training and supervision, levels of literacy, numeracy and comparing the final breath count to a gold standard.

Previously conducted studies on pneumonia diagnosis by CHWs indicate that even if CHWs are good in counting, they still often make mistakes in classifying the breath count (Kallander *et al.* 2006, Mukanga *et al.* 2011). Here, the use of beads could help in classification, and it should become clearer how for literate CHWs this potential positive effect is affected by the inaccuracy in counting when using beads. Is this because of the lack of familiarity in using the beads, or is the use of beads actually more complex and task intensive?

Concurrent to the need of more evidence regarding the utility of beads, there is a need for more research assessing the effectiveness of other devices, such as automated RR counters.

## Conclusion

Given the overall paucity of data, this review of recent studies provides insights on a range of issues to consider when implementing counting beads in iCCM programmes. This emerging evidence suggests that the introduction of well-designed ‘age-specific and colour-coded’ beads in addition to an accurate timer can help CHWs who have difficulty counting breaths and remembering age-specific cut-off rates to more accurately assess and classify fast breathing. It also has the potential to improve communication with the child’s caretakers—particularly regarding appropriate treatment options. However, more research is needed on these and other devices to decrease the inaccuracy in pneumonia diagnosis.
